# Sulfur-Containing Carotenoids from A Marine Coral Symbiont *Erythrobacter flavus* Strain KJ5

**DOI:** 10.3390/md17060349

**Published:** 2019-06-11

**Authors:** Edi Setiyono, Heriyanto, Delianis Pringgenies, Yuzo Shioi, Yu Kanesaki, Koichiro Awai, Tatas Hardo Panintingjati Brotosudarmo

**Affiliations:** 1 Ma Chung Research Center for Photosynthetic Pigments (MRCPP) and Department of Chemistry, Universitas Ma Chung, Villa Puncak Tidar N01, Malang 465151, Indonesia; edi.setiyono@machung.ac.id (E.S.); heri.yanto@machung.ac.id (H.); yshioi08@gmail.com (Y.S.); 2 Department of Coastal Resource Management, Universitas Diponegoro, Jl. Prof. Soedarto Tembalang, Semarang 50275, Indonesia; pringgenies@yahoo.com; 3 Research Institute of Green Science and Technology, Shizuoka University, 836 Ohya, Suruga-ku, Shizuoka 422-8529, Japan; kanesaki.yuh@shizuoka.ac.jp; 4 Department of Biological Science, Shizuoka University, 836 Ohya, Suruga-ku, Shizuoka 422-8529, Japan; awai.koichiro@shizuoka.ac.jp

**Keywords:** carotenoids, caloxanthin sulfate, nostoxanthin sulfate, zeaxanthin sulfate, *Erythrobacter flavus* strain KJ5

## Abstract

*Erythrobacter flavus* strain KJ5 (formerly called *Erythrobacter* sp. strain KJ5) is a yellowish marine bacterium that was isolated from a hard coral *Acropora nasuta* in the Karimunjawa Islands, Indonesia. The complete genome sequence of the bacterium has been reported recently. In this study, we examined the carotenoid composition of this bacterium using high-performance liquid chromatography coupled with ESI-MS/MS. We found that the bacterium produced sulfur-containing carotenoids, i.e., caloxanthin sulfate and nostoxanthin sulfate, as the most abundant carotenoids. A new carotenoid zeaxanthin sulfate was detected based on its ESI-MS/MS spectrum. The unique presence of sulfated carotenoids found among the currently known species of the *Erythrobacter* genus were discussed.

## 1. Introduction

Carotenoids are a family of yellow to orange-red pigments, generally comprising a 40-carbon skeleton composed of 8-isoprene units [1]. They are widely distributed in nature and more than 250 carotenoids are of marine origin [2]. The different compositions that are present in the marine microorganisms, such as microalgae and phytoplankton, has promoted the use of carotenoids as a chemical signature for rapid chemotaxonomic profiling [3,4,5,6]. In the photosynthesis process, their main functions are light harvesting and photoprotections, e.g., protection against reactive singlet oxygen and protection against blue light radiation [7]. Their wide range of properties including their beneficial effects of carotenoids on human health have drawn much attention. Carotenoids have been used as in food colorants, nutritional supplements, and for nutraceutical, cosmetic and pharmaceutical purposes [2].

The genus *Erythrobacter* was first classified by Shiba and Simidu (1982) [8]. This genus contains aerobic marine photosynthetic bacteria that have bacteriochlorophyll (BChl) *a* and carotenoids. So far 20 species are known to this *Erythrobacter* genus (*E. longus*, *E. litoralis*, *E. citreus*, *E. flavus*, *E. aquimaris*, *E. seohaensis*, *E. gaetbuli*, *E. vulgaris*, *E. nanhaesedimnis*, *E. gangjinensis*, *E. marinus*, *E. pelagi*, *E. jejuensis*, *E. odishensis*, *E. lutimaris*, *E. atlanticus*, *E. aquimixticola*, *E. arachoides*, *E. xanthus*, and *E. luteus*) [8,9,10,11,12,13,14,15,16,17,18,19,20,21,22,23,24,25,26,27]. *E. longus* was the first species of *Erythrobacter* that was identified [8]. As well as a number of non-sulfated carotenoids such as *β*-carotene, *β*-cryptoxanthin, zeaxanthin, caloxanthin, nostoxanthin, rubixanthin, bacteriorubixanthin, bacteriorubixanthinal, anhydrorhodovibrin, and spirilloxanthin [28], *E. longus* also makes sulfated carotenoids, such as erythroxanthin sulfate and caloxanthin sulfate [29]. The other *Erythrobacter* species that produces sulfur-containing carotenoid, *E. litoralis* synthesizes bacteriorubixanthinal and erythroxanthin sulfate as its major carotenoids [9]. Thus, *E. longus* and *E. litoralis* are the only members of the *Erythrobacter* genus known to have sulfur-containing carotenoids [9,29].

*Erythrobacter flavus* strain KJ5 is a yellow orange-pigmented, aerobic marine bacterium. It is ovoid rod-shaped cells of 0.1–0.5 µm × 0.2–1.0 µm dimensions (Figure S1) and has been isolated from the hard coral *Acropora nasuta*, found in the Karimunjawa Islands, Central Java province, Indonesia. Previous analysis by 16S rDNA sequence showed that this species has a similarity of 96% to *E. flavus* [30]. Recently, we have reported the complete genome sequence of this newly described bacterium [31]. In a preliminary analysis of the carotenoid composition, carotenoids, such as *β*-carotene and zeaxanthin, have been reported but there was no indication of the presence of bacteriochlorophylls [32]. However, these carotenoids are only present as minor concentration, while the major carotenoids have not yet been reported. 

Here, we report the identification of the major carotenoids of the *Erythrobacter flavus* strain KJ5 by analyses using HPLC, UV–VIS and FTIR spectrophotometers, and MS/MS with electrospray ionization. As a comparison, we also separated and identified the carotenoids from two other *Erythrobacter* species, i.e., *E. longus* and *E. nanhaesediminis.* The unique composition of sulfur-containing carotenoids from the *E. flavus* strain KJ5 was also discussed.

## 2. Results

### 2.1. Chromatography, UV–VIS and FT-IR Spectroscopy 

**Figure 1:**
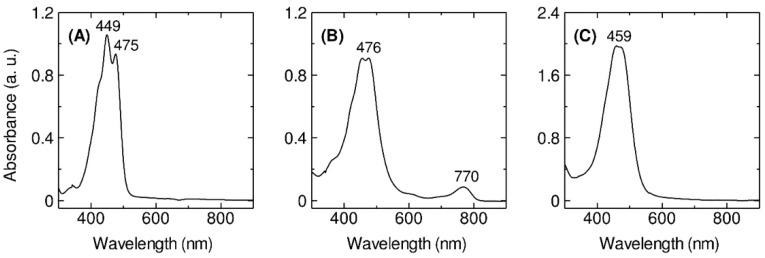
The UV–VIS–NIR absorption spectra (300–900 nm) at room temperature of the crude pigments extracted from the *E. flavus* strain KJ5 (**A**), *E. longus* (**B**), and *E. nanhaesediminis* (**C**).

The cells of *Erythrobacter flavus* strain KJ5 has been successfully grown in a Shioi liquid medium [33]. The cells were harvested after growth for 46-h culture (Figure S2) where they reached the late log phase. After the solvent extraction with a mixture of methanol and acetone (7:3, *v*/*v*), the UV–VIS absorption spectrum of the pigment extract of the bacterium (Figure 1A) was measured and compared with those spectra of the extracts from *E. longus* (Figure 1B) and *E. nanhaesediminis* (Figure 1C). The absorption spectrum of the extracted pigments exhibited typical spectral characteristics of carotenoids [1] with absorption bands in the region between 400 and 570 nm. In the case of *E. longus*, there was an absorption band at 770 nm (Figure 1B), which indicates the presence of BChl *a*, as previously reported [8]. 

**Figure 2:**
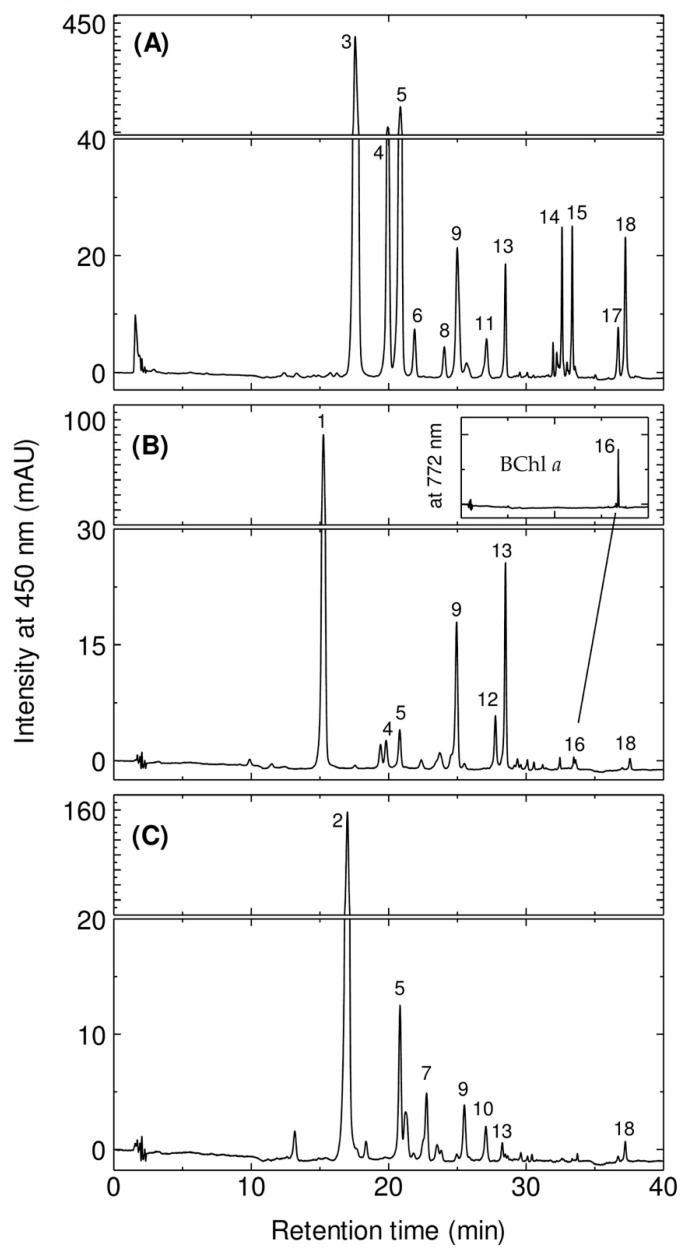
The HPLC elution profiles at λ = 450 nm of the crude pigments extracted from the *E. flavus* strain KJ5 (**A**), *E. longus* (**B**), and *E. nanhaesediminis* (**C**). Inset figure: expanded peak #16 which corresponds to BChl *a* detected at λ = 772 nm.

The HPLC elution profiles of the crude extract are shown in Figure 2. In the case of *E. flavus* strain KJ5 (Figure 2A), twelves pigments were identified by comparison with known ones from the two other species *E. longus* and *E. nanhaesediminis* (Figure 2B and Figure 2C, respectively). Assuming that the members of the genus *Erythrobacter* would employ similar carotenoid biosynthesis pathways [34,35], the peaks of *E. flavus* strain KJ5 can be tentatively assigned based on the retention times (*t*_R_) and position of maxima of their absorptions (λ_max_) as well as the spectral shape from the pigments of *E. longus* and *E. nanhaesediminis*. These chromatographic and spectrophotometric properties are compared to the references [5,8,28,29,32,36] The assignment of the pigments is listed in Table 1. 

In the case of *E. longus*, peaks #1, #9, and #13 are erythroxanthin sulfate, caloxanthin, and zeaxanthin, respectively, and they are the dominant carotenoids in the elution profile. Peak #16 (33.6 min) on the elution profile of *E. longus* is BChl *a*, while bacteriorubxanthinal and *β*-carotene were detected as peak #12 (27.8 min) and #18 (37.2 min), respectively. Caloxanthin sulfate and nostoxanthin have been reported previously in *E. longus* [28,29], and are well resolved as peaks #4 and #5 with retention times of 19.9 min and 20.8 min, respectively. In the elution profile of *E. nanhaesediminis*, two major carotenoids peaks, #2 and #5, which correspond to ketonostoxanthin and nostoxanthin, respectively, were well resolved with retention times of 17.0 and 20.8 min. Ketonostoxanthin c*is* isomer, caloxanthin, zeaxanthin, and *β*-carotene, detected as peaks #7, #9, #13, and #18, respectively, were also well resolved with retention times of 22.7, 25.0, 28.5, and 37.2 min in *E. nanhaesediminis*. Peak #10 (27.0 min) in the elution profile of *E. nanhaesediminis* could not be assigned so far, although its molecular ion estimated by MS corresponds to that of adonixanthin (Figure S4). The separation profiles of the extracted pigments of *E. longus* and *E. nanhaesediminis* were then used to assign the separated peaks of *E. flavus* strain KJ5 (Figure 2A). The first three peaks (peaks #3, #4, and #5) in the elution profile of the *E. flavus* strain KJ5 are the major carotenoids. Thus, they were assigned as nostoxanthin sulfate, caloxanthin sulfate, and nostoxanthin, respectively. Peaks #9, #13, and #18 were identified as caloxanthin, zeaxanthin, and *β*-carotene. The last peak #18 has been identified a *β*-carotene, the carotenoid that was previously reported [30,32]. 

In order to confirm the identification of the non-sulfated hydroxy carotenoids, we have used a method that calculates the linear relationship between the log capacity factor (*k’*) of the carotenoids and the number of the hydroxyl moiety of the carotenoid molecule instead of carbon atoms of the alcohol molecule [37]. It is shown in Figure S5 that the log *k’* values of *β*-carotene, *β*-cryptoxanthin, zeaxanthin, caloxanthin, and nostoxanthin were in a linear relationship as a function of the increasing number of hydroxy groups. Therefore, peak #15 (33.4 min) was assigned for the *β*-cryptoxanthin. Carotenoids of *E*. *flavus* strain KJ5 were eluted with the different *t*_R_ in their elution profile which indicated these carotenoids have the same core structure of *β*-carotene with different number of hydroxyl groups (Figure S5) [32]. The difference on the number of hydroxyl groups attached at the end rings does not effect on the spectral properties of *E*. *flavus* carotenoids, namely the same spectral shape (Figure S6) and the same λ_max_ in HPLC eluent at 452–453 nm (Table 1), indicating these carotenoids comprise of nine conjugated double bonds at the main polyene chain and two others at the cyclic end groups. Peaks #11 (27.1 mn) and #17 (36.7 min) were assigned as carotenoid *cis* isomers, i.e., zeaxanthin sulfate *cis* isomer, and *β*-carotene *cis* isomer due to the addition of *cis* band in their absorption spectra. While peak #6 (21.9 min) and #14 (32.6 min) were assigned as caloxanthin sulfate isomer and zeaxanthin isomer, respectively.

**Figure 3:**
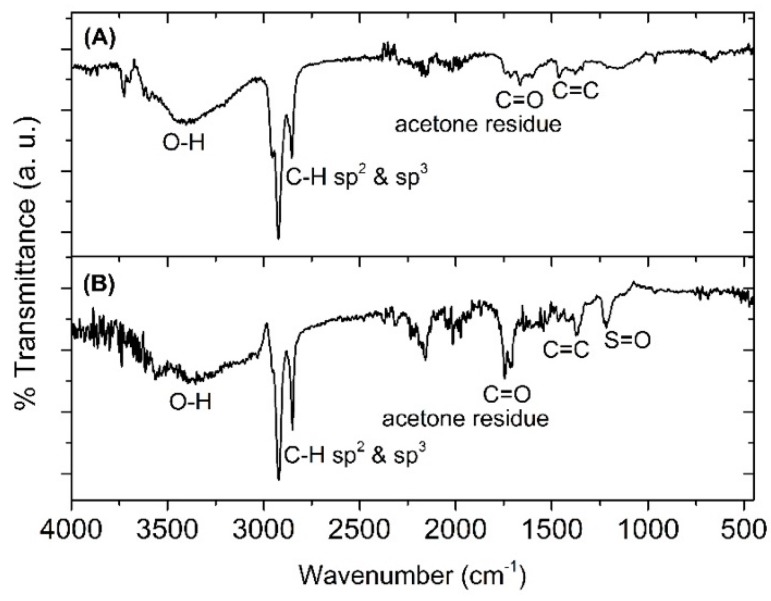
FTIR spectra of nostoxanthin (**A**) and nostoxanthin sulfate (**B**). In nostoxanthin sulfate, there is S=O sulfone stretching at 1216 cm^−1^, whereas in nostoxanthin, this was non-existent.

To confirm the structural identification of the nostoxanthin and its sulfated form, FT-IR analysis was conducted. Figure 3 shows the FT-IR spectra of the isolated nostoxanthin (A) and nostoxanthin sulfate (B), which show strong O-H stretching (3700–3100 cm^−1^), strong C-H sp^3^ and sp^2^ (3000–2750 cm^−1^), and weak C=C alkene (1375 cm^−1^). In addition, S=O sulfone stretching recorded at 1216 cm^−1^ (Figure 3B) is characteristic for a sulfate. Therefore, peak #3 (17.6 min) in the elution profile of *E. flavus* strain KJ5 was assigned as nostoxanthin sulfate.

### 2.2. Mass Spectrometry

**Figure 4:**
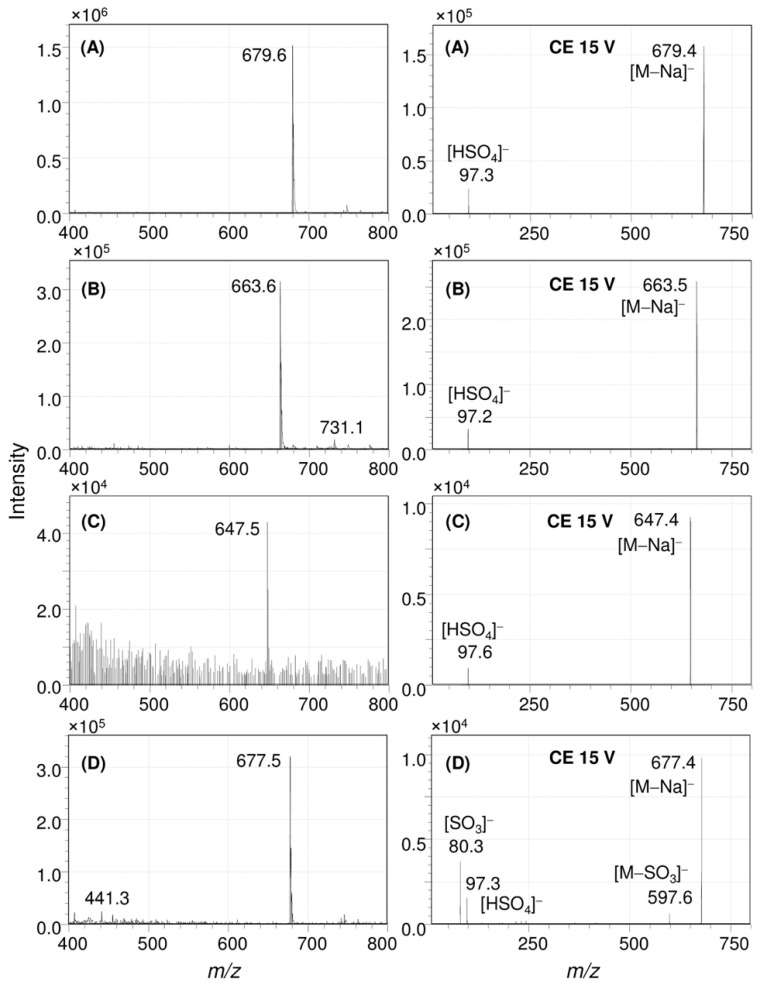
ESI-MS/MS analysis of the carotenoid sulfates in *E. flavus* strain KJ5. Full Q1 scan (**left**) and product ion scans (**right**) spectra of nostoxanthin sulfate (**A**), caloxanthin sulfate (**B**), zeaxanthin sulfate (**C**), and erythroxanthin sulfate (**D**).

In Figure 4, the result of an ESI-MS/MS analysis was conducted to understand the structure of the sulfated pigments of the *E. flavus* strain KJ5 represented by peaks #3, #4, and #8, are shown. Figure 4 left shows the full Q1 scan mass spectrum of peak #3. It has a molecular ion at mass-to-charge ratio (*m*/*z*) of 679.6 [M − Na]^−^. Further study of this product ion scan mass spectrum (right) measured using a collision energy (CE) at 15 V showed a molecular ion at *m*/*z* 679.4 [M − Na]^−^ and a fragment ion at *m*/*z* 97.3, indicating molecular ion of the sulfate group [HSO_4_]^−^. Therefore, the UV–VIS absorption (Figure S6A), FT-IR (Figure 3B), and the mass spectra (Figure 4A) of the peak #3 are consistent with the spectra of nostoxanthin sulfate as previously reported [1,36,38]. 

The mass spectrometry analysis of the isolated peak #4 from the extract of *E. flavus* strain KJ5 is shown in Figure 4B. The full Q1 scan mass spectrum showed a molecular ion at *m*/*z* 663.6 [M − Na]^−^, whilst the product ion scan also produced a fragment ion at *m*/*z* 97.2 [HSO_4_]^−^. Based on the mass spectrometry data and absorption spectrum (Figure S6B), peak #4 from the *E. flavus* strain KJ5 extract was assigned as caloxanthin sulfate, a carotenoid molecule that has also been found in *E. longus* [29]. In our *E. longus* extract, it shows at a minor component (Figure 2B). An isomer of caloxanthin sulfate was found as peak #6 at 21.9 min that has a molecular ion *m*/*z* 663.4 [M − Na]^−^ and the characteristic fragment ions at *m*/*z* 97.3 [HSO_4_]^−^. In Figure 2A, the small peak #8 showed a molecular ion at *m*/*z* 647.4 that correspond to C_40_H_55_O_5_S [M − Na]^−^ and fragment ion at *m*/*z* 97.6 (Figure 4C) for the sulfate group [HSO_4_]^−^. Therefore, peak #8 was hypothesized as a zeaxanthin sulfate. At peak #11, an isomeric form of zeaxanthin sulfate was identified at 27.1 min, which showed a molecular ion with *m*/*z* at 647.6 in the spectrum (see Figure S8B). The fragment ion at *m*/*z* 97.3, indicating molecular ion of the sulfate group [HSO_4_]^−^, was observed consistently on the spectrum of the product ion scan of another carotenoid sulfate, i.e., erythroxanthin sulfate of *E. longus* (Figure 4D right).

Absorption spectra and ESI-MS/MS analysis was used to identify peaks #5 and #9 as non-sulfated nostoxanthin and caloxanthin, Figures S6C,D and S7. Nostoxanthin (C_40_H_56_O_4_) has the presence of a molecular ion at *m*/*z* 600.5 [M]^+^ at the full Q1 scan and a fraction ion at *m*/*z* 508.6 [M − 92]^+^ (Figure S7A), while caloxanthin (C_40_H_56_O_3_) has a molecular ion at *m*/*z* 584.4 [M]^+^ and an a fraction ion at *m*/*z* 492.0 [M − 92]^+^ (Figure S7B). The results are in agreement with the previous data [36]. Other carotenoid components, i.e., peaks #13–15, #17, and #18, were well separated, purified, and identified by ESI-MS/MS (Figure S8C–G) as zeaxanthin ([M]^+^, *m*/*z* 568.5; [M − 92]^+^, 476.5); zeaxanthin isomer ([M]^+^, *m*/*z* 568.4; [M − 92]^+^, 476.6); *β*-cryptoxanthin ([M]^+^, *m*/*z* 552.4; [M − 92]^+^, 460.4); *β*-carotene *cis* isomer ([M]^+^, *m*/*z* 536.4; [M − 92]^+^, 444.4); and *β*-carotene ([M]^+^, *m*/*z* 536.5; [M − 92]^+^, 444.3), respectively. 

### 2.3. Enzyme Activity

**Figure 5:**
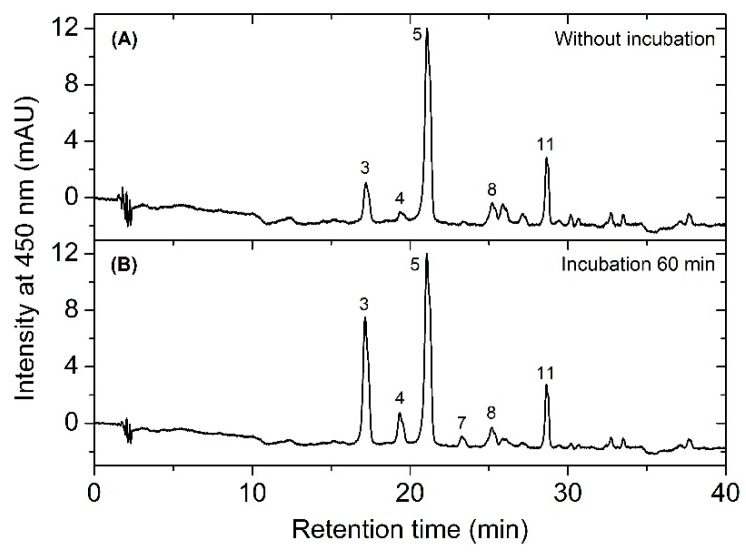
The profile of HPLC chromatograms showing the separation of carotenoids from the supernatant mixture before (**A**) and after (**B**) incubation.

We performed an enzyme activity assay to investigate the possible presence of reaction catalyzing the conversion of carotenoid into carotenoid sulfate using the cell-free extract and incubation at 30 °C for 60 min. As shown in the chromatogram in Figure 5B, after incubation the increase in peaks of caloxanthin sulfate, nostoxanthin sulfate, and also zeaxanthin sulfate was obvious, and the results further indicate that enzymes were active at this extracted condition. These data also show that zeaxanthin was converted into both nostoxanthin as well as the sulfated products. This suggests the presence of a sulfotransferase(s) in this extract. 

## 3. Discussion

In this work, we have determined the major carotenoids, including the sulfated ones, present in *E. flavus* strain KJ5 (Table 1). This was achieved by HPLC separation (Figure 2), recording absorption spectra (Figure 1 and Figure S6) and by triple quadrupole mass spectrometry with electrospray ionization (ESI-MS/MS) (Figure 4 and Figures S4, S7, and S8). The parent and product ions of the carotenoid sulfate molecules in our ESI-MS/MS measurements were detected in the negative ion mode. The product ion at *m*/*z* 97, which indicated sulfate ion [HSO_4_]^−^ could also be well recorded by the ESI-MS/MS spectra (Figure 4A). Previously, electrospray ionization has been shown to be an effective method for tracing the SO_4_^2−^ ion [39,40,41,42]. It was shown that nostoxanthin with *m*/*z* 600 [M]^+^ could lose one atom OH (*m*/*z* 17) from the 3-position, which then it was replaced by a sulfate ion group (HSO_4_^−^) resulting nostoxanthin sulfate at *m*/*z* 679.4 [M − Na]^−^ and a fragment ion at *m*/*z* 97.3. The corresponding pattern has also been observed in the case of caloxanthin and caloxanthin sulfate. In analogy, the peak #8 was suggested to be zeaxanthin sulfate molecule (*m*/*z* = 647.4 [M − Na]^−^, fragment at *m*/*z* 97.6 for [HSO_4_]^−^), produced by replacement of the OH group at the 3-position of zeaxanthin by a sulfate group. In contrast, the non-sulfated carotenoid molecules (nostoxanthin, caloxanthin, zeaxanthin, *β*-cryptoxanthin, and *β*-carotene) in *E. flavus* strain KJ5 were detected repeatedly in the positive mode and showed consistently the presence of a fragment ion at *m*/*z* [M − 92]^+^, in addition to the parent ion, [M]^+^. Previously, it was reported that most of the carotenoids produce a fragment ion at *m*/*z* [M − 92]^+^, which corresponds to a loss of toluene [43,44]. The MS/MS spectrum detected the molecular mass of the parent and daughter ions that corresponds to zeaxanthin sulfate (Figure 4C), even though it was present in very low concentrations compared to nostoxanthin sulfate and caloxanthin sulfate (Figure 2, Table 1). 

The genes involved in carotenoids biosynthesis in *E. longus* have been cloned and they were only found the *crtE*, *B*, *I*, and *Z* genes for encoding geranylgeranyl pyrophosphate synthase, phytoene synthase, phytoene desaturase, and *β*-carotene hydroxylase, respectively [36]. The genes involved in nostoxanthin biosynthesis were successfully cloned from *Brevundimonas* sp. strain SD212 [45], *Thermosynechococcus elongatus* strain BP-1 [46] and *Sphingomonas elodea* ATCC31461 [47]. The *crtG* gene encodes the 2,2′-β-hydroxylase (CrtG) that is responsible for converting zeaxanthin into its 2-hydroxylated (caloxanthin) and 2,2′-dihydroxylated (nostoxanthin) products, which are structurally rare xanthophylls [45,46,47]. The genes encoding for the incorporation of sulfate to the 3-hydroxy group of nostoxanthin, caloxanthin, and eventually zeaxanthin, remains unexplored. 

The presence of sulfated natural products compound in marine organisms is not surprising. Sulfur has been known as the fourth most abundant element by importance in seawater after chlorine, sodium, and magnesium. The sulfate ion is the most stable state of sulfur in seawater and is the second most abundant anion by importance after chloride. More than 500 sulfated compounds have been identified in marine organisms [48]. There has also been numerous investigations related to the antithrombotic, antifouling, antiviral, and anti-inflammatory activities displayed by these sulfated compounds [49,50,51,52,53]. Hydrogen sulfide is toxic to a wide range of eukaryotic organisms, such as coral and sponges, and acts by inhibiting cytochrome c oxidase and hemolymph catalase activity [54,55]. Hydrogen sulfide has also led to the initiation of coral black band disease [56]. Previous studies have suggested that the co-existence of sulfur-oxidizing bacteria might convert hydrogen sulfide to sulfate, thereby contributing to coral health, including corals such as those from the genus *Acropora* [48,57,58,59,60,61,62]. 

The 20 species of the *Erythrobacter* genus have been isolated from different hosts. *E. longus* was isolated from high-tidal seaweed [8]. *E. vulgaris* was isolated from the starfish *Stellaster equestris* [15]. *E. litoralis*, *E. citeus*, *E. flavus*, *E. gangjinensis*, *E. marinus*, *E. pelagi*, *E. jejuensis*, and *E. xanthus* were isolated from seawater [9,10,11,17,18,19,20,26]. *E. aquimixticola* has habitat in the place where the ocean and a freshwater spring meet [25], while *E. aquamaris*, *E. seohaensis*, *E. gaetbuli*, *E. luteolus*, *E. nanhaisediminis*, *E. odishensis*, *E. lutimaris*, *E. atlanticus*, and *E. luteus* were isolated from various sediments and soil [12,13,14,16,21,22,23,24]. One other, *E. arachoides*, was isolated from a glacier on the Tibetan Plateau [27]. However, it was only *E. longus* and *E. litoralis* that were reported to produce new carotenoids, i.e., with the sulfate group at the carotenoid molecule [9,29]. In this study, we found that *E. flavus* strain KJ5 also produced sulfur-containing carotenoids, such as caloxanthin sulfate and nostoxanthin sulfate, as the most abundant carotenoids. In addition, a unique carotenoid, zeaxanthin sulfate, was also identified for the first time based on its ESI-MS/MS analysis. Further study to understand how this bacterium metabolizes sulfated carotenoids and its function is currently on-going.

## 4. Materials and Methods 

### 4.1. Bacteria and Cell Growth

*Erythrobacter flavus* strain KJ5 was obtained from the Laboratory of Tropical Marine Biotechnology, Diponegoro University (Semarang, Indonesia). This bacterium was first isolated from a hard coral *Acropora nasuta*, from the Karimunjawa Islands. The complete genome sequence of the bacterium has been reported [31], whereas *E. longus* (JCM6170) and *E. nanhaesediminis* (JCM16125) were provided by the RIKEN BRC through the National Bio-Resource Project of the MEXT, Japan.

The bacterial cells were grown in a Shioi liquid medium as [33], containing 34.3 mM NaCl, 24.5 mM MgCl_2_·6H_2_O, 14.1 mM Na_2_SO_4_, 6.71 mM KCl, 3.40 mM CaCl_2_·2H_2_O, 2.38 mM NaHCO_3_, 4.08 mM ferric citrate, yeast extract 2.0 g/L (*w*/*v*), polypeptone 1.0 g/L (*w*/*v*), casamino acids 1.0 g/L (*w*/*v*), and glycerol 1.0 mL (*v*/*v*). The cells were incubated at 28 °C in a shaking incubator (New Brunswick Scientific Excella E24, Edison, NJ, USA) under the dark conditions for 80 h. The cells were harvested after a 46-h culture where they reach late log phase. These growth conditions are where nostoxanthin sulfate and caloxanthin sulfate reach an optimum concentration (Figure S2B). The cells were harvested by centrifugation at 10,000 rpm for 20 min at 4 °C and the collected cells were stored at −30 °C until used. Experiments were performed at least three times using independent cell cultures, and the average and SE are shown.

### 4.2. Carotenoids Extraction

The collected cells were homogenized in a mixture of methanol and acetone (7:3, *v*/*v*; 1 mL mixture per 0.1 g cells) by vortexing for three times (1 min vortex, 1 min in ice bath), and then disrupted by sonication at a pulse mode with 60% amplitude and 10-s on/30-s off for 10 min (QSonica, Newtown, CT, USA). The pigment extract was centrifuged at 8000× *g* for 2 min to obtain the supernatant. The resulting extract was dried at 35 °C using a rotary evaporator (Heidolph Laborota 4010 digital, Schwabach, Germany) at 100 rpm connected to a Huber minichiller and vacuum pump and N_2_ gas. The crude pigment extract was stored at −30 °C until use.

### 4.3. HPLC Analysis

The carotenoids were separated and purified by a preparative-HPLC (Shimadzu preparative-UFLC, Kyoto, Japan) using a Symmetry C_8_ column (150 × 4.6 mm, 3.5 μm particle size, 100 Å pore size) (Waters, Milford, MA, USA) with two eluents as the mobile phase. Eluent A was composed of 50% methanol and 25% acetonitrile, as well as 25% pyridine solution (0.25 M, pH 5) (*v*/*v*) and Eluent B was composed of 20% methanol, 60% acetonitrile, and 20% acetone. The used solvents (HPLC grade; MERCK, Darmstadt, Germany) were degassed for 5 min with ultrasonication prior use. The carotenoid separation was performed by using the following gradient: 15% of eluent A for the first 4 min and then changed to 100% of eluent A. Total separation time was 30 min and was conducted at flow rate of 1 mL/min. The pure carotenoids were stored in a deep freezer at −85 °C until use.

The determination of the different carotenoids was realized via an analytical-HPLC (Shimadzu analytical-UFLC, Kyoto, Japan). The column and eluent are same as those used in preparative HPLC. However, the method used refers to the following published method by Zapata et al. (2000) [5]. The gradient elution were 100% of eluent A from 0–22 min, changed to 40% eluent B at min 22–28, 5% eluent A (min 28–38), and 100% eluent A at min 40–50 at a flow rate of 1 mL/min with the column oven temperature set at 30 °C. The total separation time was 50 min. The carotenoids were detected with a diode array detector (Shimadzu SPD M20A, 190–800 nm) at 450 nm. 

The standard carotenoids, *β*-carotene and zeaxanthin were obtained from DHI LAB (Horsholm, Denmark) and NATChrom (Malang, East Java, Indonesia). *β*-cryptoxanthin was highly purified from peels of Japanese citrus unshu mikan (*Citrus unshiu*).

### 4.4. Absorption and FTIR Spectroscopy Measurement

Absorption spectra of the carotenoids were measured by a UV–VIS 1700 spectrophotometer (Shimadzu) at room temperature. The characterization of the carotenoids was based on their absorption spectrum of the pigment extracts. The dried pigment extracts and pure carotenoids were diluted in 1 mL methanol and their spectra measured at wavelengths (λ) of 200–1100 nm. Identification of the pigments was based on their spectral properties and λ at maximum absorbance (λ_max_) as well as their comparative study to other references. FT-IR measurements of the purified carotenoids were performed with a Jasco FT/IR-6800 (Tokyo, Japan), by using the ATR (Diamond) method. The type of ATR used was the Jasco ATR Pro One. The pure carotenoids were diluted in acetone and dropped into the sample chamber and left until the solvent evaporated. Thereafter, 64 scans were accumulated with ranges from 400–4000 cm^−1^. The spectrum resolution was 4 cm^−1^. The ATR prism was ZnSe. The measurement was conducted at room temperature under dark conditions. Subsequently, the data obtained were analyzed using OriginPro 8.5.1 software (OriginLab, Northampton, MA, USA).

### 4.5. MS/MS Analysis

The UFLC XR Prominence coupled with a LCMS-8030 triple quadrupole mass spectrometer (Shimadzu) was used for the identification of the carotenoids. The analysis was conducted through a Symmetry C_8_ column (150 × 4.6 mm, 3.5 μm particle size, 100 Å pore size) (Waters, Milford, USA) with gradient elution using two solvents: solvent A (H_2_O with 0.1% formic acid), and solvent B (methanol with 0.1% formic acid) at a flow rate of 1.5 mL/min. Each run of the LC-MS/MS was performed with isocratic elution with 90% solvent B for 2 min. The column oven temperature was 30 °C, the DL temperature was 250 °C, the nebulizing gas flow rate was 3 L/min, the heat block temperature was 400 °C, the drying gas flow rate was 15 L/min, the mass range was from 400 to 700 *m*/*z*, the cooler temperature was 5 °C, and the electrospray was in its ionization (ESI) mode. Optimization of the collision energy (CE) was carried out by multiple reaction monitoring (MRM) to find the optimal CE. The data were used for carotenoid species analysis. Identification of the pure carotenoids was based on the precursor ion, product ion, and single ion monitoring (SIM) data.

### 4.6. Pigment Identification

The identification of pigments was carried out by the HPLC and MS/MS analyses according to the chromatographic, i.e., retention time (t*_R_*), spectrophotometric, i.e., the spectral shape and maxima absorption wavelength (λ_max_), and mass, i.e., precursor and fragment ions, properties compared to the literatures [5,28,29,34,36]. Carotenoids in *E. flavus* were compared to those from the different *Erythrobacter* species such as *E. longus* whose structures are already reported [28,29], with the methods previous described [6]. The carotenoids identified from *E. longus*, i.e., erythroxanthin sulfate, caloxanthin sulfate, nostoxanthin, caloxanthin, and bacteriorubixanthinal, were isolated individually with min. 97% purity and were used as standard carotenoids. The data consisted of a full Q1 and product ion scans at optimized CE of each stand carotenoid were stored in the LabSolution MS Library (Shimadzu). Then, the pigments in cell extracts of were identified by comparison of chromatographic and spectral data recorded with those stored in the library, using LabSolution LCMS Ver. 5.4 (Shimadzu). The software compared retention times and aligned MS/MS data, to calculate a match factor and produced a degree of similarity between spectra. In addition, the complete genome sequence of the bacterium was used to predict the carotenoid biosynthetic pathway in as reported previously [63]. FT-IR measurement of sulfated-carotenoids was done by focusing on the presence of the S=O sulfone stretching vibration. 

### 4.7. Assay of the Enzyme Activity

To determine the presence of any enzyme activity able to convert carotenoids into carotenoid sulfate compounds, the following modified method was used [64]. Cells (0.1 g) were lysed by a sonication for 5 min (60% amplitude, 30-s on, 10-s off) using 1.0 mL of buffer containing 0.1 M KH_2_PO_4_ pH 7.0 with 0.05 mM KCl and 0.24% (*v*/*v*) Triton X-100 at 5 °C. After centrifugation at 15,000× *g* for 15 min, the supernatant was used for the enzyme assay. To start the enzymatic reaction, the supernatant was incubated for 60 min at 30 °C. Thereafter, the supernatant was mixed with 1.0 mL methanol and acetone (7:3, *v*/*v*) solvent, and then 0.5 mL diethyl ether was added. Next, the extracted pigments in the diethyl ether layer were dried using the flow of N_2_ gas. The dried sample was then ready for HPLC analysis using the method described above in Section 4.3. 

## Figures and Tables

**Table 1:** Identification of the carotenoids in three different *Erythrobacter* species. Strain 1, *E. flavus* strain KJ5; 2, *E. longus*; 3, *E. nanhaesediminis*. +, detected and −, not detected. The chemical structures of the major carotenoid identified are presented in Figure S3.

Peak No	Identification	*t*_R_ (min)	λ_max_ (nm)	Capacity Factor (*k*’)	Molecular Ion	Fragment Ion	Ref.	Strain		
								1	2	3
1	Erythroxanthin sulfate	15.3	464	8.71	677.5 [M − Na]^−^	80.3 [SO_3_]^−^, 97.3 [HSO_4_ ]^−^, 597.6 [M − SO_3_]^−^	[29,36]	−	+	−
2	Ketonostoxanthin	17.0	463, 475	9.83	614.6 [M]^+^	596.0 [M − 18]^+^	[36]	−	−	+
3	Nostoxanthin sulfate	17.6	(427), 452, 480	10.18	679.6 [M − Na]^−^	97.3 [HSO_4_]^−^	[36]	+	−	−
4	Caloxanthin sulfate	19.9	(427), 453, 480	11.69	663.6 [M − Na]^−^	97.2 [HSO_4_]^−^	[29,36]	+	+	−
5	Nostoxanthin	20.8	(427), 452, 480	12.27	600.5 [M]^+^	508.6 [M − 92]^+^	[28,36]	+	+	+
6	Caloxanthin sulfate isomer	21.9	(427), 453, 480	12.94	663.4 [M − Na]^−^	97.3 [HSO_4_]^−^	−	+	−	−
7	Ketonostoxanthin c*is* isomer	22.7	353, 454, (472)	13.46	614.5 [M]^+^	596.4 [M − 18]^+^, 582.4 [M − 32]^+^	−	−	−	+
8	Zeaxanthin sulfate	24.0	(427), 453, 481	14.31	647.5 [M − Na]^−^	97.6 [HSO_4_]^−^	−	+	−	−
9	Caloxanthin	25.0	(427), 453, 480	14.92	584.4 [M]^+^	492.0 [M − 92]^+^	[28,36]	+	+	+
10	Unidentified	27.0	468	16.20	582.5 [M]^+^	536.5 [M − 46]^+^, 490.3 [M − 92]^+^	−	−	−	+
11	Zeaxanthin sulfate *cis* isomer	27.1	331, (427), 452, 476	16.27	647.6 [M − Na]^−^	97.2 [HSO_4_]^−^	−	+	−	−
12	Bacterio- rubixanthinal	27.8	510	16.68	596.5 [M]^−^	550.4 [M − 46]^−^	[28,36]	−	+	−
13	Zeaxanthin	28.5	(426), 453, 478	17.15	568.5 [M]^+^	476.5 [M − 92]^+^	[5,28,32]	+	+	+
14	Zeaxanthin isomer	32.6	(426), 453, 479	19.77	568.5 [M]^+^	476.6 [M − 92]^+^	−	+	−	−
15	*β*-cryptoxanthin	33.4	(426), 453, 479	20.24	552.5 [M]^+^	460.4 [M − 92]^+^	[28,36]	+	−	−
16	BChl *a*	33.6	362, 601, 769	20.39	911.3 [M]^+^	783.5 [M − 128]^+^	[8]	−	+	−
17	*β*-carotene *cis* isomer	36.7	340, (425), 449, 474	22.37	536.1 [M]^+^	444.4 [M − 92]^+^	−	+	−	−
18	*β*-carotene	37.2	(426), 452, 478	22.71	536.5 [M]^+^	444.3 [M − 92]^+^	[5,28,32]	+	+	+

## Supplementary Materials

The following are available online at https://www.mdpi.com/1660-3397/17/6/349/s1, Figure S1: The morphological image of the *E. flavus* strain KJ5 cell that was observed by using a JEOL JEM 1400 transmission electron microscope at 100 KV with magnification at 20,000×, Figure S2: The growth curve of *E. flavus* strain KJ5 in Shioi liquid medium (A) and relative concentration of carotenoids during growth phase (B), Figure S3: Chemical structure of the carotenoids from *E. flavus* strain KJ5, Figure S4: Analysis of keto-carotenoids and bacteriochlorophyll in *E. longus* and *E. nanhaesediminis* using ESI-MS by the Q1 scan (left) and product ion scan (right), Figure S5: The plot of log *k’* value of *β*-carotene (*k’* = 1.36), *β*-cryptoxanthin (*k’* = 1.31), zeaxanthin (*k’* = 1.23), caloxanthin (*k’* = 1.17), and nostoxanthin (*k’* = 1.09) versus the number of hydroxyl moieties of the carotenoid molecule showed a linear line with *R*^2^ = 0.9948, Figure S6: The UV–VIS absorption spectra of nostoxanthin sulfate (A); caloxanthin sulfate (B); nostoxanthin (C); and caloxanthin (D) in *E. flavus* strain KJ5, Figure S7: Analysis of the non-carotenoids sulfate in *E. flavus* strain KJ5 using ESI-MS/MS by Q1 scan (left) and product ion scan (right), nostoxanthin (A) and caloxanthin (B)., Figure S8: Analysis of the carotenoids in the *E. flavus* strain KJ5 using ESI-MS by Q1 scan (left) and product ion scan (right).
