# Supplementary material for: Sulfur-Containing Carotenoids from A Marine Coral Symbiont Erythrobacter flavus Strain KJ5
Source: Mar Drugs. 2019 Jun 11;17(6):349. doi: 10.3390/md17060349 (PMC6627997; doi:10.3390/md17060349)
Supplement: Supplementary file 1 [file marinedrugs-17-00349-s001.pdf]

marine drugs

Supplementary Materials: Sulfur-containing carotenoids from a marine coral symbiont *Erythrobacter flavus* strain KJ5

Edi Setiyono [1], Heriyanto [1], Delianis Pringgenies [2], Yuzo Shioi [1], Yu Kanesaki [3], Koichiro Awai [4] and Tatas Hardo Panintingjati Brotosudarmo [1,*]

[1] Ma Chung Research Center for Photosynthetic Pigments (MRCPP) and Department of Chemistry, Universitas Ma Chung, Villa Puncak Tidar N01, Malang 465151, Indonesia
[2] Department of Coastal Resource Management, Universitas Diponegoro, Jl. Prof. Soedarto Tembalang, Semarang 50275, Indonesia
[3] Research Institute of Green Science and Technology, Shizuoka University, 836 Ohya, Suruga-ku, Shizuoka 422-8529, Japan
[4] Department of Biological Science, Shizuoka University, 836 Ohya, Suruga-ku, Shizuoka 422-8529, Japan
* Correspondence: tatas.brotosudarmo@machung.ac.id; Tel.: +62-341-550-171

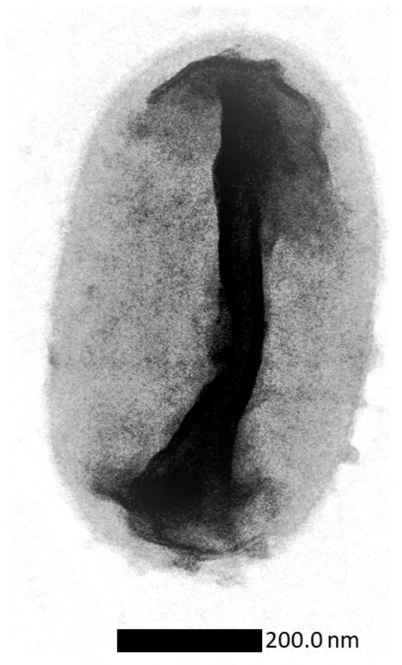

Figure S1. The morphological image of the *E. flavus* strain KJ5 cell that was observed by using the Transmission Electron Microscope JEOL JEM 1400 at 100 KV with magnification at 20000×. Cell was an ovoid rod-shaped, non-flagellated, sized at a 0.1–0.5 μm width and 0.2–1.0 μm length.

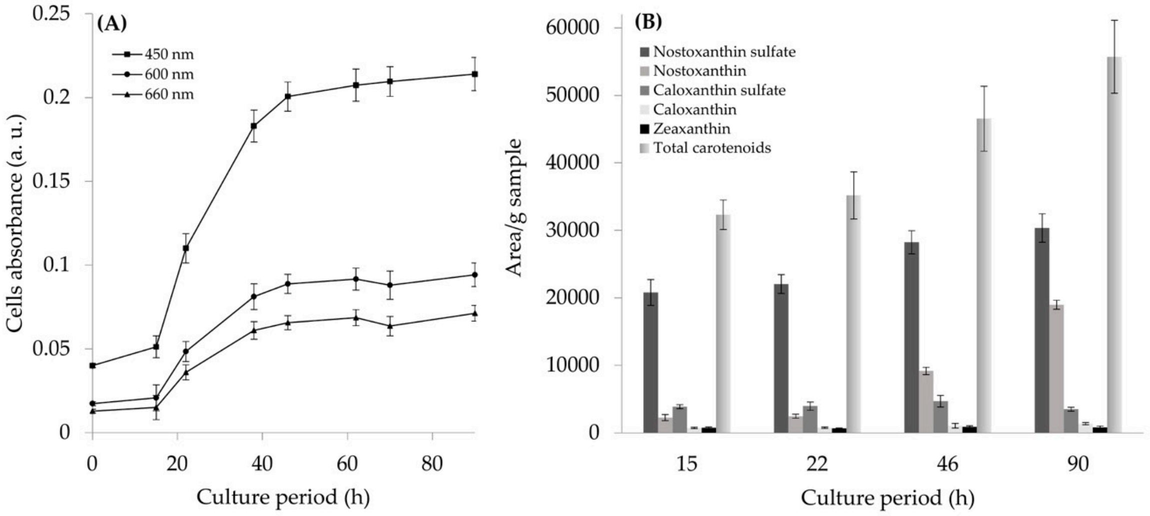

Figure S2. The growth curve of *E. flavus* strain KJ5 in Shioi liquid medium (A) and relative concentration of carotenoids during growth phase (B). The cells were grown aerobically on a shaker (100 rpm) for 3 days. The growth reached to the exponential phase after a 40-h culture. The results are averages of at least three independent experiments, and the vertical bar represents SE.

1. *β*-carotene

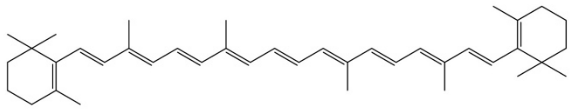

2. *β*-cryptoxanthin

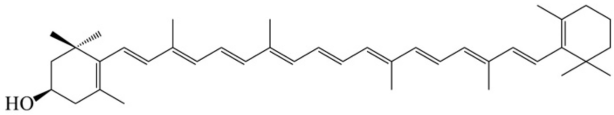

3. Zeaxanthin

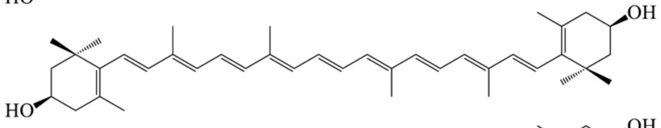

4. Caloxanthin

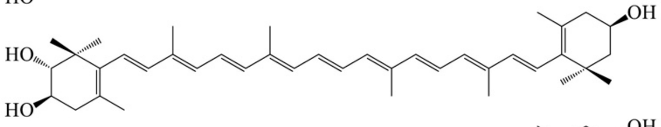

5. Nostoxanthin

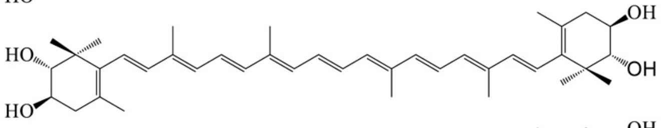

6. Zeaxanthin sulfate

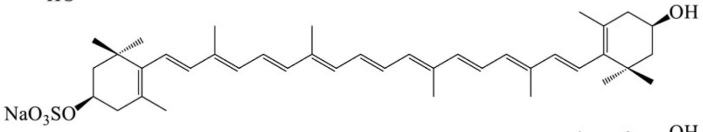

7. Caloxanthin sulfate

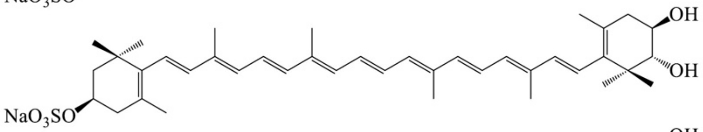

8. Nostoxanthin sulfate

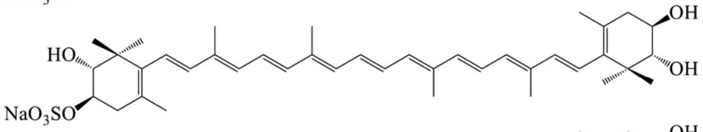

9. Erythroxanthin sulfate

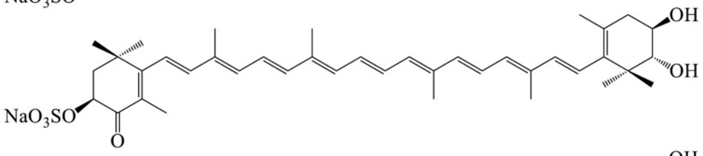

10. Ketonostoxanthin

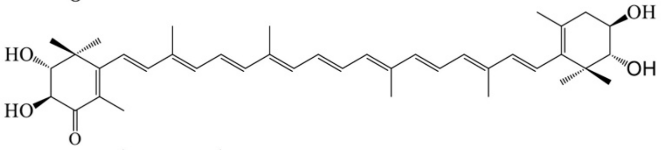

11. Bacteriorubixanthinal

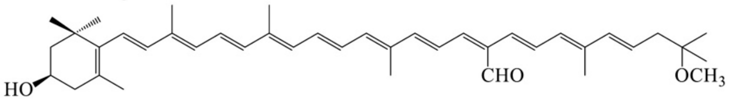

Figure S3. Chemical structure of the major carotenoids identified in Table 1.

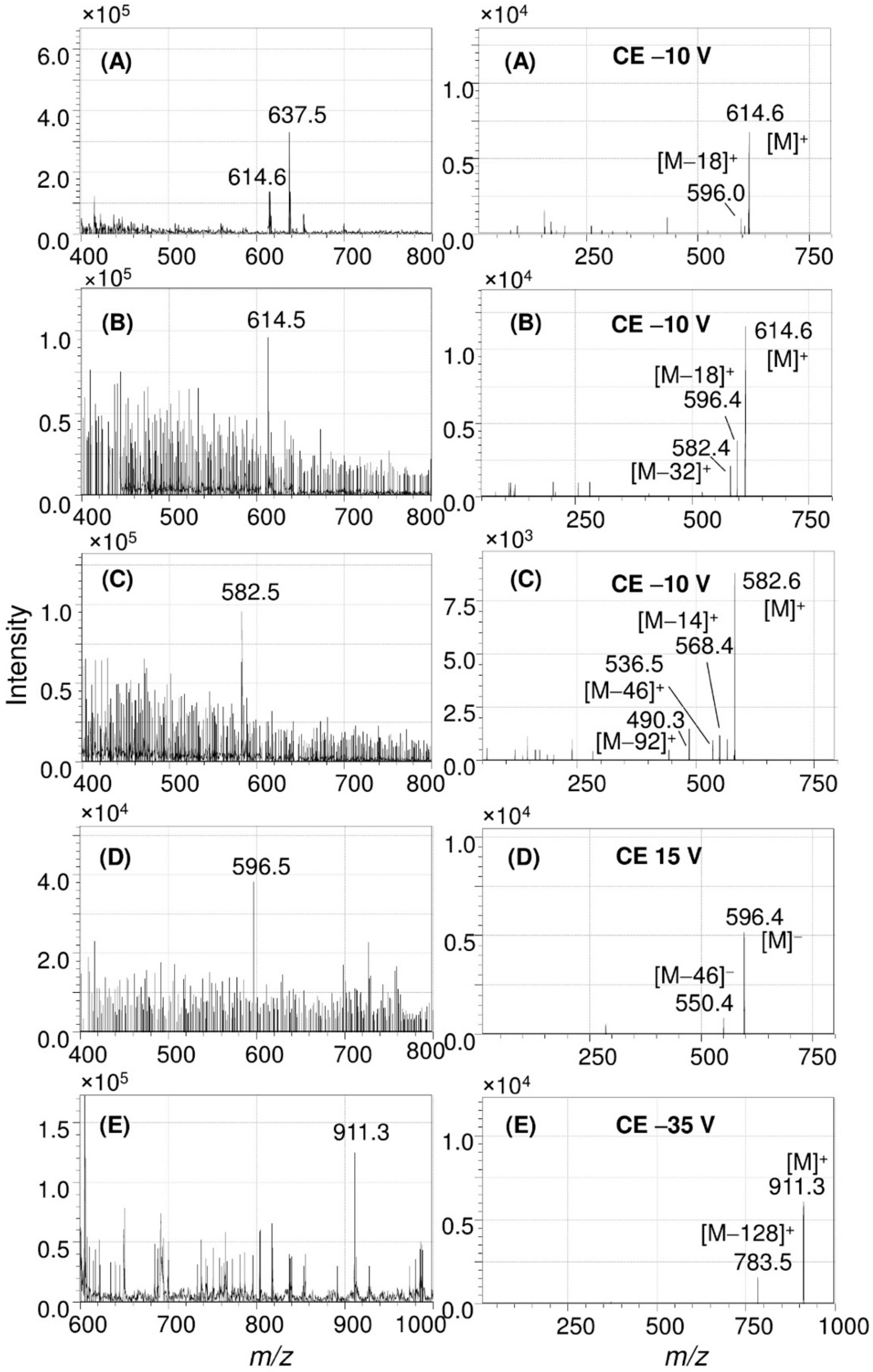

Figure S4. Analysis of keto-carotenoids and bacteriochlorophyll in *E. longus* and *E. nanhaesediminis* by ESI-MS by Q1 scan (left) and product ion scan (right). Ketonostoxanthin (A); Ketonostoxanthin *cis* isomer (B); Unidentified (C); bacteriorubixanthinal (D); and bacteriochlorophyll *a* (E)

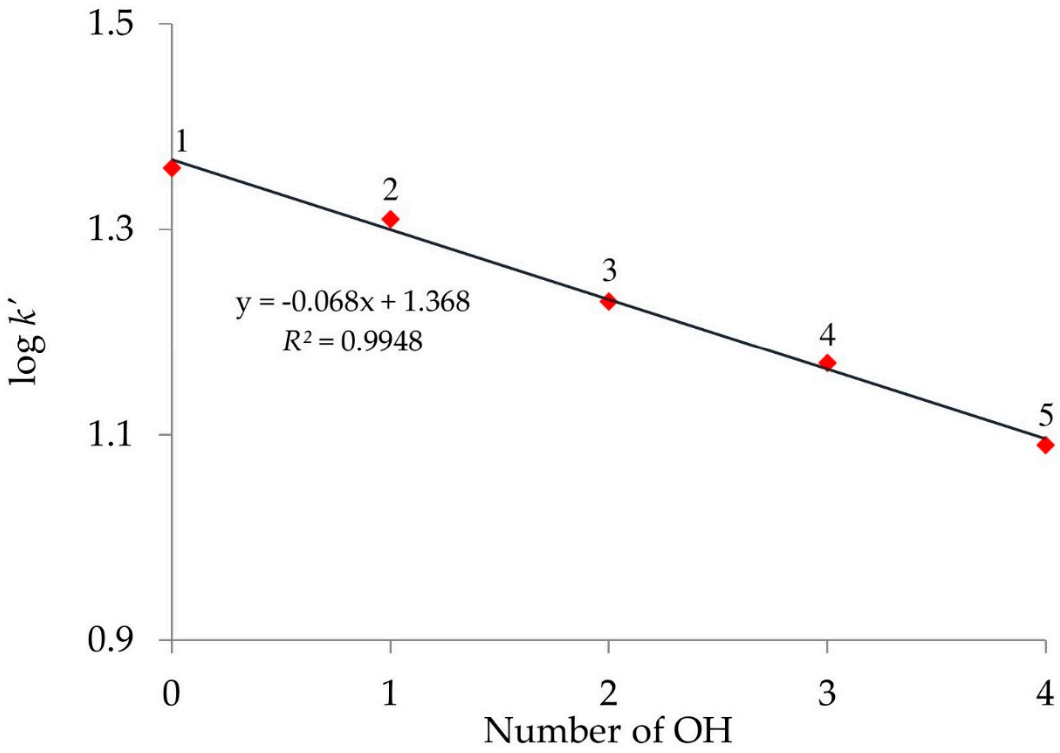

Figure S5. The plot of log k' value of β-carotene (k' = 1.36), β-cryptoxanthin (k' = 1.31), zeaxanthin (k' = 1.23), caloxanthin (k' = 1.17) and nostoxanthin (k' = 1.09) versus number of hydroxyl moiety of the carotenoid molecule showed a linear line with R^2 = 0.9948. The capacity factor (k') was measured by $k' = (t_R - t_0)/t_0$, where t_R and t_0 are the retention times of the retained and unretained solute in the given system, respectively. 1. β-carotene (None); 2. β-cryptoxanthin (OH = 1); 3. Zeaxanthin (OH = 2); 4. Caloxanthin (OH = 3); 5. Nostoxanthin (OH = 4).

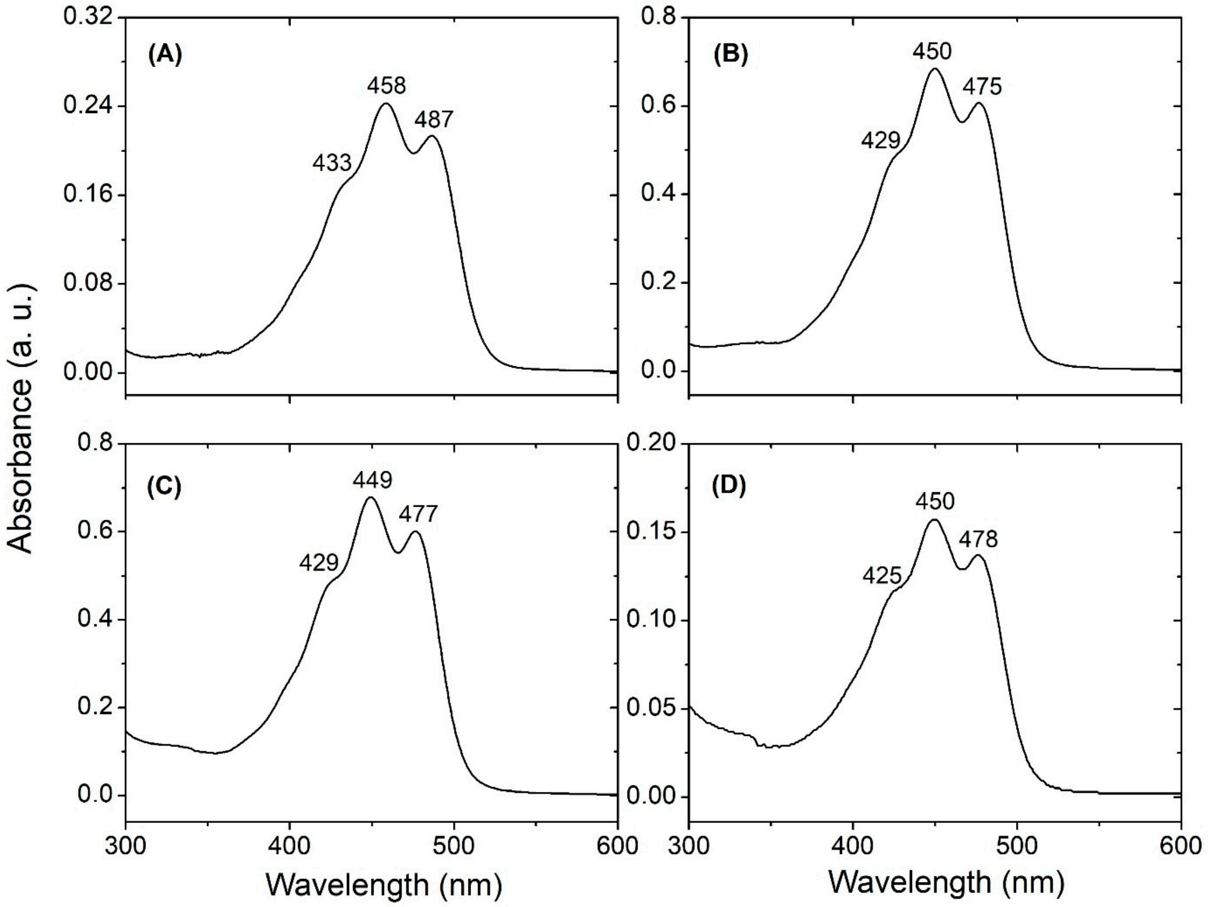

Figure S6. The uv-vis absorbtion spectra of nostoxanthin sulfate in chloroform:MeOH (2:1, v/v) (A) and caloxanthin sulfate in MeOH (B); as well as nostoxanthin (C) and caloxanthin in EtOH (D) of *E. flavus* strain KJ5.

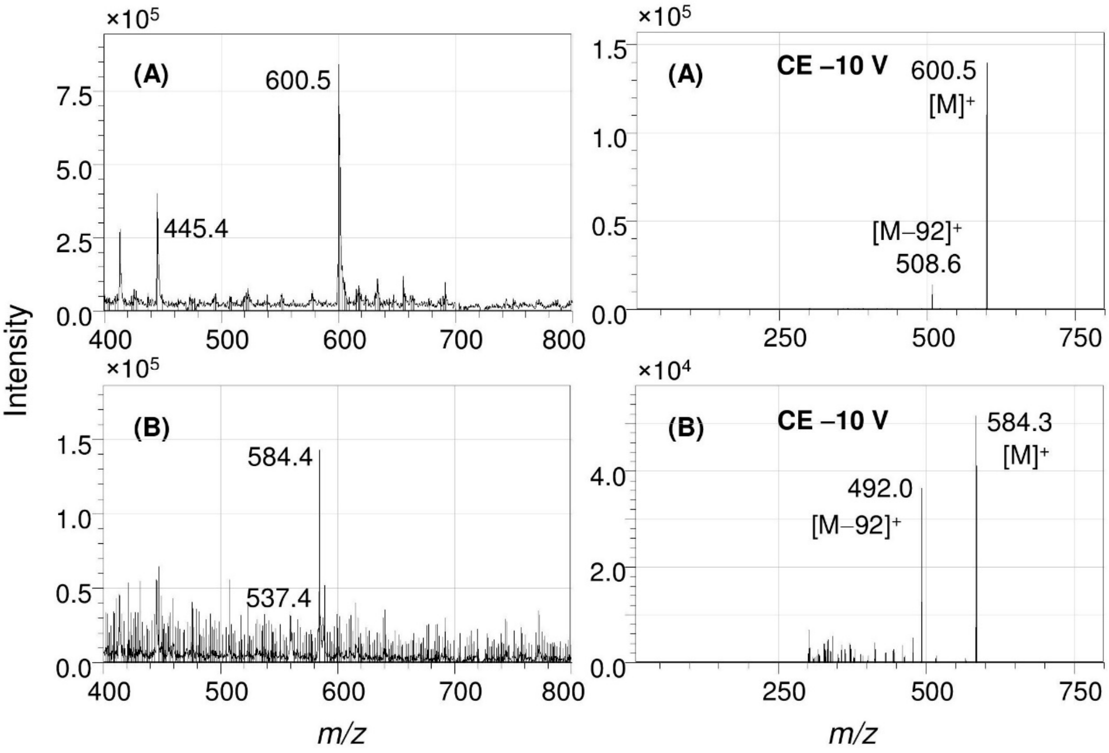

Figure S7. Analysis of the non-carotenoids sulfate in *E. flavus* strain KJ5 by ESI-MS/MS by Q1 scan (left) and product ion scan (right), nostoxanthin (A) and caloxanthin (B).

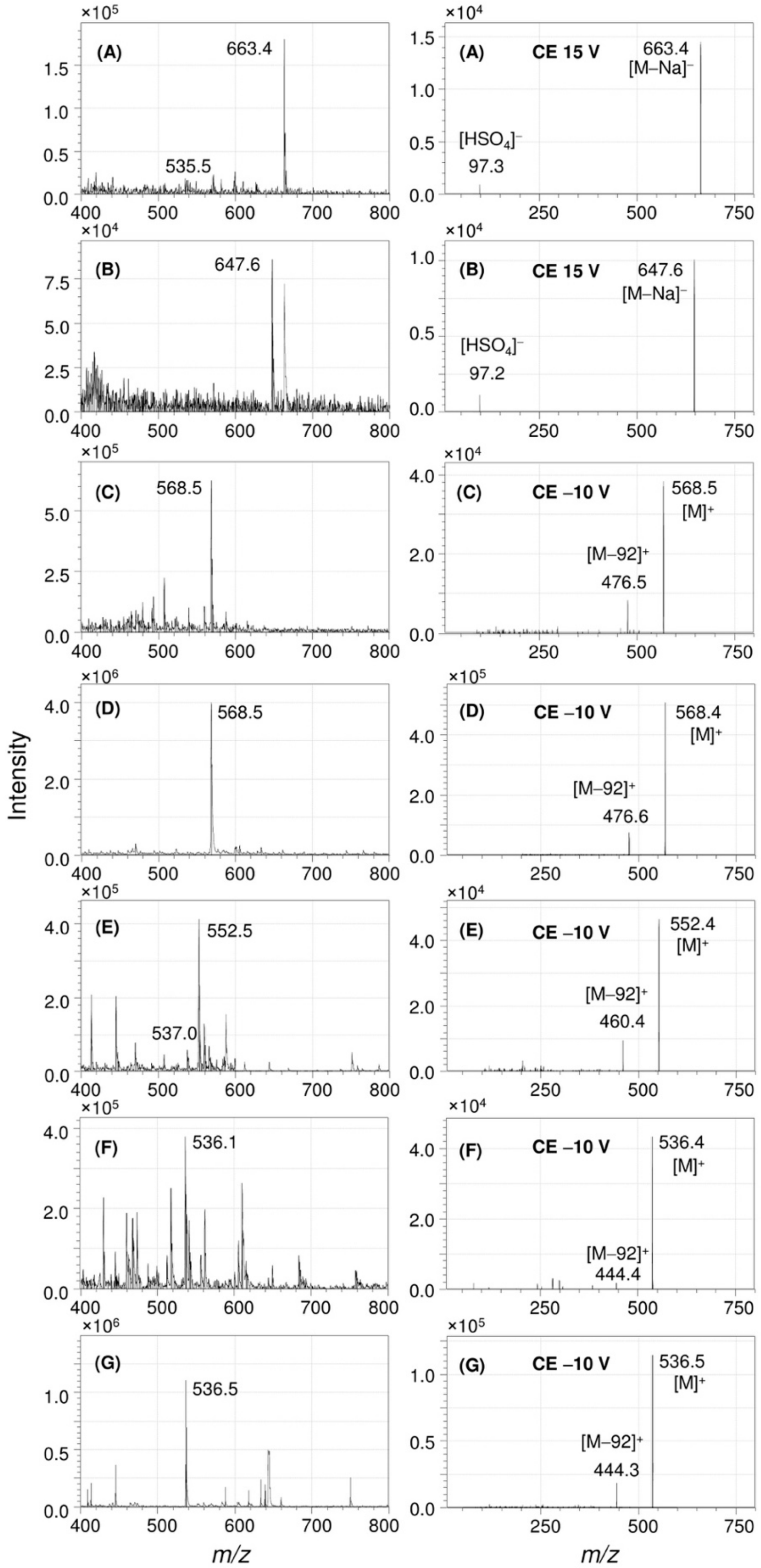

Figure S8. Analysis of the carotenoids in *E. flavus* strain KJ5 using ESI-MS by the Q1 scan (left) and product ion scan (right). Caloxanthin sulfate isomer (A); zeaxanthin sulfate *cis* isomer (B); zeaxanthin (C); zeaxanthin isomer (D); β-cryptoxanthin (E); β-carotene *cis* isomer (F); and β-carotene (G).

© 2018 by the authors. Submitted for possible open access publication under the terms and conditions of the Creative Commons Attribution (CC BY) license (http://creativecommons.org/licenses/by/4.0/).
